# No link between viral findings in the prostate and subsequent cancer development

**DOI:** 10.1038/sj.bjc.6603480

**Published:** 2006-11-21

**Authors:** J Bergh, I Marklund, C Gustavsson, F Wiklund, H Grönberg, A Allard, O Alexeyev, F Elgh

**Affiliations:** 1 Department of Medical Biosciences/Pathology, Umeå University, Umeå S-90185, Sweden; 2 Department of Clinical Microbiology/Virology, Umeå University, Umeå S-90185, Sweden; 3 Department of Radiation Sciences/Oncology, Umeå University, Umeå S-90185, Sweden; 4 Department of Medical Epidemiology and Biostatistics, Karolinska Institute, PO Box 281, Stockholm SE-171 77, Sweden

**Keywords:** DNA virus, *C. albicans*, prostate, benign prostate hyperplasia

## Abstract

In an investigation of 201 prostate tissue samples from patients with benign prostate hyperplasia that later progressed to prostate cancer and 201 matched controls that did not, there were no differences in the prevalence of adenovirus, herpesvirus, papilloma virus, polyoma virus and *Candida albicans* DNA.

Mutations in genes associated with the immune defence have been identified in hereditary prostate cancer, indicating that infection and/or inflammation of the prostate may be important mediators for the development of prostate cancer (Palapattu *et al*, 2005; Sun *et al*, 2005). Moreover, population studies have revealed an increased relative risk for development of prostate cancer in men with a prior history of sexually transmitted infections (Dennis and Dawson, 2002). These findings support the hypothesis that an infectious agent can be a potential cofactor in prostate cancer development. Human papilloma virus (HPV), Epstein–Barr virus (EBV) and the polyoma viruses JCV and BKV represent viruses with proven linkage to different human cancers and have been traced in prostate cancer tissues (Grinstein *et al*, 2002; Zambrano *et al*, 2002). To further evaluate if a viral infection could contribute to prostate cancer development, we conducted a case–control study of 402 patients with benign prostate hyperplasia (BPH), of which 201 later progressed to prostate cancer. We examined whether the presence of genetic traces of EBV, herpes simplex virus (HSV) 1 and 2, cytomegalovirus (CMV), adenovirus, HPV, polyoma viruses BKV and JCV and *Candida albicans* in the prostate correlate with histological inflammation and subsequent prostate cancer diagnosis.

## MATERIALS AND METHODS

A case–control study of 402 archival prostate tissue samples obtained during transurethral resection of the prostate (TURP) collected at the Department of Pathology at the University Hospital of Northern Sweden, Umeå was conducted as described previously (Alexeyev *et al*, 2006; Bergh *et al*, 2006). Briefly, tissues were obtained from men with BPH (median age 64, range 51–71), fixed in formalin, paraffin-embedded and stored at room temperature until tested. A total of 201 men developed prostate cancer at least 6 months after the TURP. For each case, a control was randomly selected from a cohort of patients that did not develop prostate cancer. The case–control pairs were matched for year of birth, residence and year of TURP. Histological inflammation was graded as mild or severe as described (Alexeyev *et al*, 2006). DNA from prostate tissue was purified and checked for integrity as described (Alexeyev *et al*, 2006). Nested PCR assays were used for all the assays except HPV and *C. albicans* PCRs. Primers and PCR protocols for adenovirus (Allard *et al*, 2001), CMV (Brytting *et al*, 1991), EBV (Meyohas *et al*, 1996), HSV1 and 2 (Aurelius *et al*, 1991) and HPV (de Roda Husman *et al*, 1995) were used with minor modifications. Primers for the polyoma viruses JCV and BKV and *C. albicans* were designed according to published sequence information (Table 1). To verify the positive PCR findings, PCR products were purified with QIAquick Purification Kit protocol (Qiagen^®^, Hilden, Germany) and directly sequenced in the ABI PRISM 3700 DNA ANALYSER (AME Bioscience, Toroed, Norway) using the Big Dye™ Terminator Cycle Sequencing kit 1.1 (Applied Biosystems, Forster City, CA, USA). Histological inflammation in prostate tissue was graded as described earlier (Alexeyev *et al*, 2006). Fisher exact test was used for statistical analysis.

## RESULTS

Out of 402 samples tested, 352 (87.6%) were positive for the human *β*-globin gene. These samples were considered to have sufficient DNA quality and were therefore used for subsequent analysis in viral and fungal PCRs. Of the 352 samples tested, 31 (8.8%) were positive for EBV and 10 (2.8%) for JCV. No other viral DNAs were detected. Of 240 samples that were available for *C. albicans*-specific PCR, two were (0.8%) positive. We then assessed whether the detection of EBV, JCV and *C. albicans* in the prostate correlates with subsequent prostate cancer diagnosis. In total, 159 matched case–control pairs with complete information on EBV and JCV and 115 pairs with complete information for *C. albicans* were available. Of the 29 positive EBV samples, 15 (9.4%) were in the case group (progressed to cancer) and 14 (8.8%) were in the control group. Of 10 positive JCV samples, three (1.9%) were in the case group and seven (4.4%) were in the control group. The two samples scored positive for *C. albicans* were found in the case group. There was no difference in the occurrence of severe inflammation in EBV- or JCV-positive samples *vs* virus-negative samples (data not shown).

## DISCUSSION

To the best of our knowledge, this is the first study investigating the presence of eight different DNA viruses and *C. albicans* in a large series of men with BPH. Owing to the case–control design of the present study, it had a potential to evaluate if viral/fungal infection could precede and, possibly, contribute to prostate inflammation and prostate cancer development. Only archival samples positive for the *β*-globin gene were subsequently tested for microbial DNAs, thus ensuring good quality DNA and absence of PCR inhibitors. Of eight DNA viruses tested, only EBV and JCV were found in the prostate tissue. This observation is in accord with previous studies (Grinstein *et al*, 2002; Zambrano *et al*, 2002). These viruses are unlikely to contribute to prostate cancer development in the patients studied owing to the similar occurrence in the case and control groups. Data on the presence of HPV in benign and malignant prostate tissues are contradictory. Some groups have reported high rates of detection (Noda *et al*, 1998; Serth *et al*, 1999; Zambrano *et al*, 2002), whereas others have not found HPV (Effert *et al*, 1992; Strickler *et al*, 1998). All samples tested in this study were negative for HPV, thus making it an unlikely contributing factor for subsequent cancer development in the 352 patients studied. Our study did not find any association between the presence of EBV and JCV and histological inflammation in the prostate. These viruses are therefore unlikely as triggering factors of chronic prostate inflammation. In conclusion, our study has shown that the prostate can harbour mixed microbial communities. Epstein–Barr virus, JCV and *C. albicans* do not appear to contribute to chronic prostate inflammation and subsequent prostate cancer development.

## Figures and Tables

**Table 1:** Oligonucleotide primer sequence for polyoma viruses JC virus and BK virus and *C. albicans*

**Microorganism**	**Position**	**Sequence**	**Amplimer size (nt)**
JC virus	2656–2677^a^	5′ TGC AGT TTT CCT GTG TGT CTG C 3′	259
	2914–2893	5′ TTT AGG CCA GTT GCT GAC TTT G 3′	
	2722–2743	5′ CAG TGC TTG ATC CAT GTC CAG A 3′	167
	2888–2867	5′ TGC CAT TCA TGA GAG GAT TGT G 3′	
			
BK virus	1452–1472^b^	5′ GAA AAA ACT ATT GCC CCA GGA G 3′	192
	1643–1625	5′ AGT TTT GGC ACT TGC ACG G 3′	
	1487–1508	5′ AAC TGC TCC TCA ATG GAT GTT G 3′	114
	1600–1579	5′ CCC CTG GAC ACT CTC CTT TTC T 3′	
			
*C. albicans*	5.8S gene	5′ GCC TGT TTG AGC GTC GTT TC 3′	82
		5′ CTA CCG TCT TTC AAG CAA ACC C 3′	

a Sequence positions refer to the JC virus isolate SK-6.

b Sequence positions refer to the BK virus Dunlop strain sequence.
